# Enzymatic Cleavage of 3’-Esterified Nucleotides Enables a Long, Continuous DNA Synthesis

**DOI:** 10.1038/s41598-020-64541-z

**Published:** 2020-05-05

**Authors:** Shiuan-Woei LinWu, Ting-Yueh Tsai, Yu-Hsuan Tu, Hung-Wen Chi, Yu-Ping Tsao, Ya-Chen Chen, Hsiang-Ming Wang, Wei-Hsin Chang, Chung-Fan Chiou, Johnsee Lee, Cheng-Yao Chen

**Affiliations:** 1 Personal Genomics, Inc., Zhubei, Hsinchu, 30261 Taiwan; 2 Department of Medical Laboratory Science and Biotechnology, College of Medicine, National Cheng Kung University, Tainan City, 70101 Taiwan; 3 Institute of Biological Chemistry, Academia Sinica, Nankang, Taipei 115 Taiwan

**Keywords:** Biochemistry, Biotechnology, Structural biology

## Abstract

The reversible dye-terminator (RDT)-based DNA sequencing-by-synthesis (SBS) chemistry has driven the advancement of the next-generation sequencing technologies for the past two decades. The RDT-based SBS chemistry relies on the DNA polymerase reaction to incorporate the RDT nucleotide (NT) for extracting DNA sequence information. The main drawback of this chemistry is the “DNA scar” issue since the removal of dye molecule from the RDT-NT after each sequencing reaction cycle leaves an extra chemical residue in the newly synthesized DNA. To circumvent this problem, we designed a novel class of reversible (2-aminoethoxy)-3-propionyl (Aep)-dNTPs by esterifying the 3’-hydroxyl group (3’-OH) of deoxyribonucleoside triphosphate (dNTP) and examined the NT-incorporation activities by A-family DNA polymerases. Using the large fragment of both *Bacillus stearothermophilus* (BF) and *E. coli* DNA polymerase I (KF) as model enzymes, we further showed that both proteins efficiently and faithfully incorporated the 3’-Aep-dNMP. Additionally, we analyzed the post-incorporation product of N + 1 primer and confirmed that the 3’-protecting group of 3’-Aep-dNMP was converted back to a normal 3’-OH after it was incorporated into the growing DNA chain by BF. By applying all four 3’-Aep-dNTPs and BF for an *in vitro* DNA synthesis reaction, we demonstrated that the enzyme-mediated deprotection of inserted 3’-Aep-dNMP permits a long, continuous, and scar-free DNA synthesis.

## Introduction

The next-generation sequencing (NGS) technologies have revolutionized modern biomedical research. The sequencing data output, scale, and speed of NGS enable researchers to address a diverse range of biological questions ranging from the analysis of genome-wide rare somatic mutations, structural variations, epigenetic modifications to the study of microbial diversity in humans and in the environments^1–6^. Nowadays, emerging NGS applications are rapidly developed and applied for disease diagnosis in the clinical laboratories^7–9^. Although the formats and specifications of today’s commercial DNA sequencers are different, the core of these robust sequencing platforms are mainly powered by the DNA sequencing-by-synthesis, or SBS, chemistry. The SBS chemistry relies on the DNA polymerase reaction to incorporate deoxyribonucleotides for extracting DNA sequence information. The fundamental differences amongst current NGS technologies are the types of modified nucleotides and their compatible DNA polymerases used in the sequencing reaction^10^.

**Figure 1:**
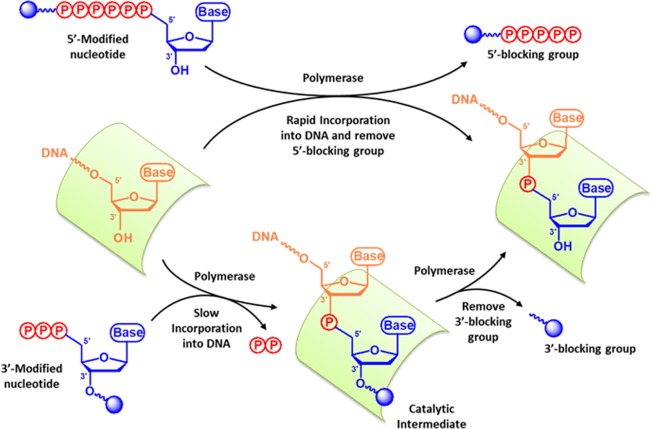
Uninterrupted, scar-free DNA sequencing-by-synthesis reaction using 5’ or 3’-modified nucleotides. Upper reaction pathway, the hexaphosphate-linker-dye moiety is attached to the terminal phosphate of the nucleotide (NT). The removal of pentaphosphate-linker-dye moiety by DNA polymerase during the NT-incorporation will leave a normal 3’-OH group on the primer terminus (**one-reaction step**), which permits further nucleotide incorporations by DNA polymerase. Lower reaction pathway, an ester-linker-dye group is conjugated via the 3’-O-ribose of the nucleotide. After the NT-incorporation by DNA polymerase, the 3’-modified group on the primer terminus is concurrently edited by DNA polymerase and converted back to a regular 3’-OH, which allows a continuous DNA synthesis (two-reaction steps).

Except for the pyrosequencing^11,12^ and semiconductor-based proton-sequencing techniques^13^, in which both directly use the normal nucleoside triphosphate (dNTP) for DNA sequencing reactions, the nucleotide (NT) substrates adopted by the mainstream SBS-driven NGS platforms, including Illumina, Qiagen, and Pacific Biosciences (PacBio), are extensively modified. In both Illumina’s and Qiagen’s sequencing chemistry, the purine (A or G) or pyrimidine (C or T) base of nucleotide (NT) is individually attached with a distinct spectrum of fluorescent dye for signal detection. Moreover, the regular 3′-hydroxyl group (3’-OH) on the deoxyribose of each NT is substituted with a protecting, chemical group^14–18^. Unlike the normal dNTPs, the incorporation of these 3’-protected NTs by DNA polymerase (Pol) terminates the DNA-chain elongation since these NTs lack a functional 3’-OH for DNA extension^15,18,19^. The 3’-protecting group on the primer terminus of incorporated nucleotide (N + 1) can be subsequently removed to restore a regular 3’-OH of deoxyribose. As a result, the next cycle of NT addition by DNA Pol can resume and the growing DNA chain can further be extended. However, in this reversible SBS reaction, the removal of fluorescent dye from the NT after each reaction cycle leaves an extra, chemical linker moiety, which covalently connects the dye molecule to the base, on the normal purine or pyrimidine NTs in the newly synthesized DNA. These molecular scars on the nascent DNA strain may perturb the subsequent protein-DNA interaction and affect the performance of DNA Pol in the later cycles of DNA sequencing reactions^10,20,21^. To avoid the scare issue, the fluorescent molecule with a unique, optical spectrum can be directly attached to the 5’-terminal γ-phosphate of each NT, respectively, as exemplified by the PacBio’s real-time SBS scheme ^17,22^. Because the phosphoryl transfer (PT) reaction catalyzed by DNA Pol occurs between the 3′-OH of primer terminus and α-phosphate of incoming NT^23,24^, at the end of each NT-incorporation cycle results in one base addition to the primer terminus (N + 1) and a free dye-labeled polyphosphate group^17,22^. Hence, the outcome of each NT addition leaves no molecular vestige on the newly synthesized DNA, and the N + 1 primer terminus possesses no blocking group to hinder the following rounds of DNA elongation. Consequently, the DNA sequencing reaction can continue uninterruptedly (Fig. 1**, Upper reaction pathway**).

**Figure 2:**
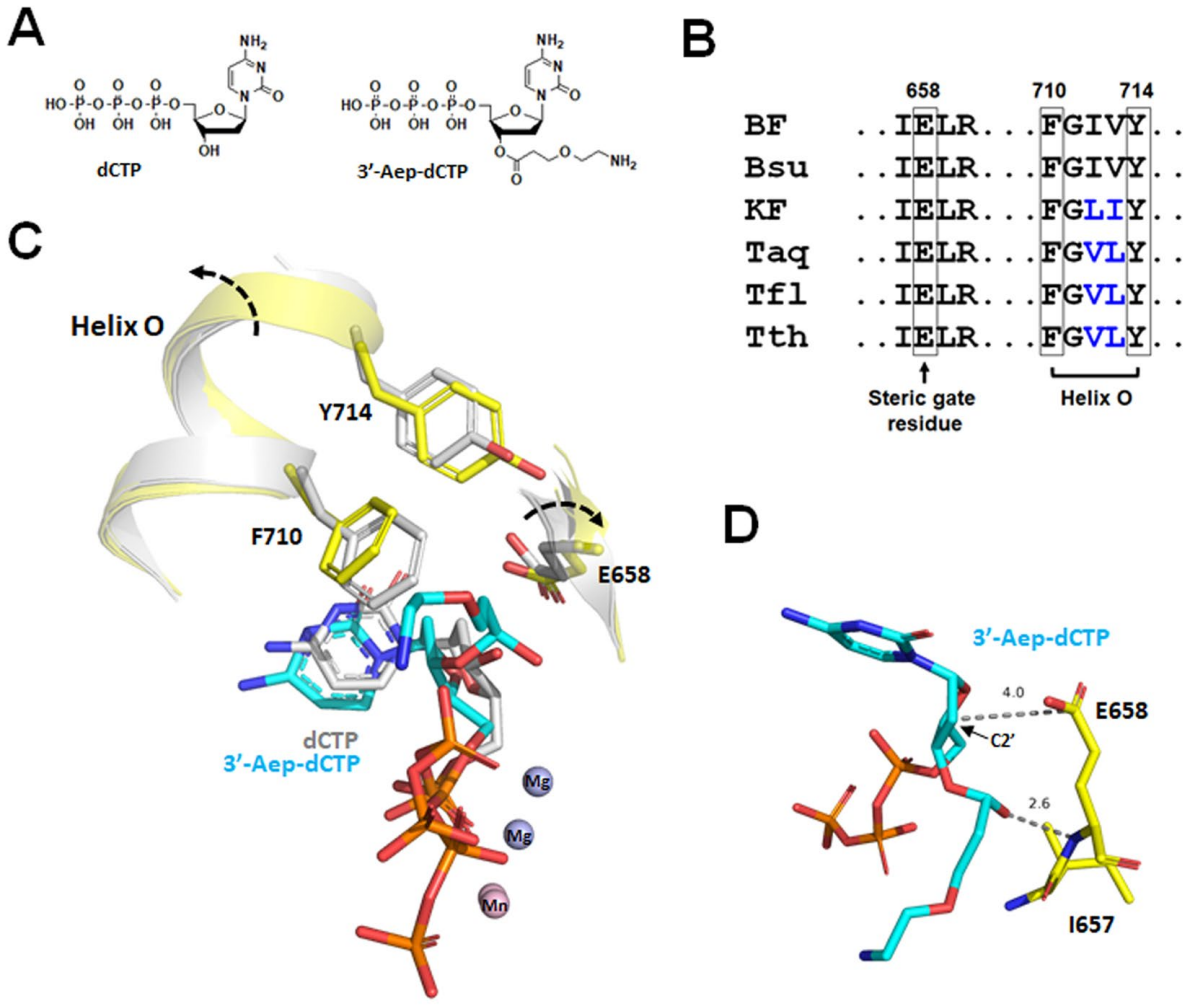
Accommodation of 3’-esterified dCTP by BF. (**A**) Structures of dCTP (Left) and (2-aminoethoxy)-3-propionyl (Aep)-dCTP (Right). (**B**) The conserved steric gate residue and helix O of commercially available A-family DNA polymerases. (**C**) The structural model of 3’-Aep-dCTP in the nucleotide-binding pocket of BF. (**D**) Interaction of the steric gate residue E658 with 3’-Aep-dCTP inside the nucleotide-binding pocket of BF. Direct hydrogen-bonding interactions are shown as black dashed lines, and the distances between C2’ position and E658 are also illustrated.

Alternatively, to deal with the molecular remnant problem after the elimination of fluorescent dye from the NTs in the reversible dye-terminator (RDT) chemistry, the direct attachment of dye to the 3’-OH of deoxyribose has been proposed^25,26^. In this reaction scheme, the insertion of NT with a 3’-fluorescent tag (3’-FT) to a DNA primer prevents the DNA Pol from further extension due to the lack of a 3’-OH at the N + 1 primer end. Like the common RDT-NT, the elimination of 3’-FT from the N + 1 primer terminus restores a normal 3’-OH of the deoxyribose. Hence, the nascent DNA strand can once again be elongated by DNA Pol, and the sequencing reactions can be performed continuously (Fig. 1**, Bottom reaction pathway**). Unfortunately, this approach is difficult to implement since the NT with a bulky, chemical substitution on the 3′-OH of NT is generally a poor substrate for DNA Pols^10,27^. This type of 3’-modified NT imposes a strong steric hindrance on the nucleotide-binding pocket (NBP) of DNA Pol, and therefore decreases the NT-binding affinity and the overall NT-incorporation efficiency of enzyme^27,28^. Unexpectedly, the 3’-esterified 2’-deoxynucleoside 5’-monophosphate seems to be tolerated and incorporated by both Taq and the proofreading-deficient T7 DNA polymerase (Sequenase)^25,26,29^. These two A-family DNA polymerases are able to utilize the 2’-deoxyl-3’-anthranyloyl-dNTPs as substrates for DNA synthesis^29^. However, it remains elusive whether it is a common feature for other A-family DNA polymerases (AF-DNAPs) to accommodate such a large, ester modification at the 3’-OH position on the deoxyribose of NT. Also, how well AF-DNAPs utilize this class of 3’-esterified dNTPs have not been evaluated. Furthermore, the feasibility of applying these 3’-esterified dNTPs for a scar-free, continuous DNA synthesis reaction has not been tested. In this study, we utilize a novel class of reversible 3’-esterified dNTPs (Fig. 2A, 3**’-Aep-dNTP**) and examine the NT-incorporation activities of these modified nucleotides by A-family DNA polymerases. Also, using the large fragment of *Bacillus stearothermophilus* DNA polymerase I (BF) as a model enzyme, we tested the feasibility of applying these 3’-esterified dNTPs for an uninterrupted and scar-free DNA synthesis.

**Figure 3:**
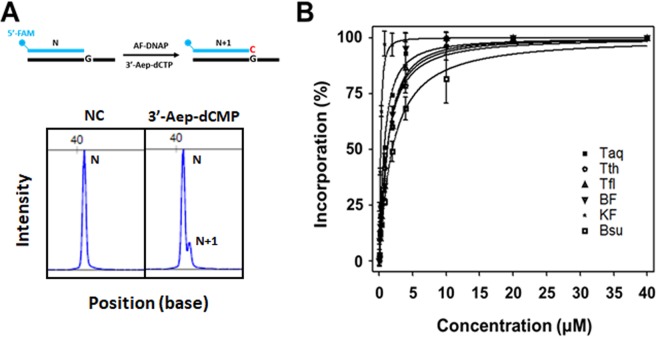
Incorporation of 3’-Aep-dCMP by commercially available A-family DNA polymerases (AF-DNAPs). (**A**) Top, the schematic representation of single 3’-Aep-dCMP incorporation by Taq, Tth, Tfl, BF, KF, or Bsu. Bottom, DNA fragment analysis of the primer (N) and the primer plus an incorporated 3’-Aep-dCMP by BF (N + 1). (**B**) Activities of single 3’-Aep-dCMP incorporation by Taq, Tth, Tfl, BF, KF, or Bsu, respectively. The primer-extension assays were performed as described in the Methods using 0.1, 0.2, 0.4, 0.8, 2, 4, 10, 20, or 40 μM of 3’-Aep-dCTP in the reaction.

## Results

### Accommodation of 3’-esterified dNTP by the large fragment of *Bacillus stearothermophilus* DNA polymerase I (BF)

To elucidate the intrinsic features of A-family DNA polymerases (AF-DNAPs) and deduce how AF-DNAPs could readily accept and incorporate the 3’-esterified nucleotide (NT), we first built the computational model of BF ternary complex with a primer-template DNA and a 3’-esterified nucleotide based on the existing ternary structure of BF (PDB file:1LV5)^31^ (Fig. 2C). We chose BF for structural simulation because of its high degree of homology to other AF-DNAPs. From the protein sequence alignment of three, large fragments of DNA polymerase I from *Bacillus stearothemophilus* (BF), *Thermus aquaticus* (KlenTaq), and *Escherichia coli* (Klenow Fragment, KF) shows that BF shares a 56% and 55% of identity with KlenTaq and KF, respectively (data not shown). In addition to the sequence and structure conservation of BF with other A-family DNA polymerases, there are abundant crystal structures of BF available in the Protein Data Bank (PDB), which make the outcome of computational models more predictable.

To construct the model of BF ternary complex in conjunction with a primer-template DNA and a 3’-esterified dNTP, we directly replaced the natural dCTP with a modified (2-aminoethoxy)-3-propionyl (Aep)-dCTP in the closed conformation of BF ternary structure containing a primer-template DNA and a dCTP. The (2-aminoethoxy)-3-propionyl group was selected for its flexibility and availability to attach a fluorescent dye (Fig. 2A). As depicted in the Fig. 2C, the 3’-ester group of 3’-Aep-dCTP faces inward and adjacent to the E658 residue of BF, which was previously shown to exclude the C2’ modifications on the deoxyribose in the nucleotide-binding pocket of BF^31^. Meanwhile, the (2-aminoethoxy)-3-propionyl group of 3’-Aep-dCTP protrudes toward the pocket opening and the three negatively charged phosphates of the nucleotide remain associated with two divalent cations (Mg^2+^ or Mn^2+^ in the Fig. 2C). The superimposition of model with the original BF ternary complex reveals the prominent “exclusion effect” of 3’-Aep-dCTP within the nucleotide-binding pocket of BF. The larger size of 3’-Aep-dCTP causes a significant rotation of E658 residue and, therefore, increases the distance between the side-chain carboxylate of E658 and the C2’ position of 3’-Aep-dCTP from a 3.1 Å to ~4.0 Å. The reposition of E658 side-chain forms an alternative hydrogen bond with the 3’-ester carbonyl group of 3’-Aep-dCTP (Fig. 2D), which potentially stabilizes the 3’-Aep-dCTP within the nucleotide-binding pocket (NBP) of BF. Accordingly, the finger subdomain of BF also reorients in order to make room for the 3’-Aep-dCTP within the NBP. The O-helix of BF finger subdomain rotates and shifts both aromatic side-chains of F710 and Y714 residues away from the 3’-Aep-dCTP (Fig. 2C). The reorientations of F710 and Y714 side chains slightly open up the O-helix of finger subdomain and create more space for the (2-aminoethoxy)-3-propionyl group to fit inside the NBP of BF. Altogether, the synergistic movements of E658 sugar-steric gate and F710 and Y714 residues on the conserved O-helix of BF finger subdomain seem to play a critical role in accepting a 3’-Aep-dCTP. Interestingly, the E658, F710, and Y714 residues of BF are highly conserved among other A-family DNA polymerases (Fig. 2B). Hence, it is likely that these A-family DNA polymerases may all be able to incorporate the 3’-esterified dNMPs.

### Incorporation of 3’-Aep-dNMP by BF and other A-family DNA polymerases

To test whether BF and other commercially available A-family DNA polymerases (KF, Taq, Tth, Tfl, and Bsu) may efficiently incorporate the 3’-esterified dNMPs during DNA replication, we synthesized all four bases of 3’-Aep-dNTPs (Fig. 2A) according to the previously published procedures^30,32^. The molecular mass and identity of synthetic 3’-Aep-dNTPs were measured and confirmed by the electrospray-ionization mass spectrometry (ESI/MS) (Figure S1). The purity and stability of nucleotides were further determined by the analytical HPLC. All four 3’-Aep-dNTPs show a purity, ranging from 99.32% to 99.92%, and a stability from 98.75% to 99.57% after incubation at 60 °C for 1 hour (Figure S2).

To determine the incorporation activity of 3’-Aep-dNMP by BF, the primer-extension assays were performed, and the reaction products were separated by the capillary electrophoresis as described in the Methods. The fluorescent intensity of primer (N) and N + 1 nucleotide bands were measured by the DNA fragment analysis. As illustrated in the Fig. 3A, the primer (N) and the N + 1 nucleotide (NT) bands show distinct peaks in the DNA fragment analysis, respectively. The relative NT-incorporation activity (% of N + 1 products) of BF can then be calculated as described in the Methods. As shown in the Fig. 3B, the incorporation activity of 3’-Aep-dCMP by BF shows a nucleotide-concentration dependence. Increasing concentrations of 3’-Aep-dCTP from 0.125, 0.25, 0.5, 1, 2, 4, 10, 20 to 40 μM in the DNA polymerization reaction correspond to higher NT-incorporation activities of BF (inverted, filled triangles). The steady-state *Michaelis* constant (*K*_m_) of 3’-Aep-dCTP for BF can be determined as described in the Methods. The result was listed in the Table 1. The *K*_m_ value of BF for the 3’-Aep-dCTP was estimated to be 1.50 ± 0.02 μM. Likewise, the *K*_m_ values for five other commercially available A-family DNA polymerases (KF, Taq, Tth, Tfl, and Bsu) were also measured by the same experimental procedures. As shown in the Fig. 3B, except for the KF (dots), the Taq, Tth, Tfl, and Bsu DNA polymerases show a similar degree of NT-incorporation activity (filled squares, open circles, filled triangles, and open squares, respectively). KF exhibits higher NT-incorporation activities across a series of 3’-Aep-dCTP concentrations than other DNA polymerases. As a result, the *K*_m_ value of 3’-Aep-dCTP for KF is 0.28 ± 0.01 μM, which is much lower than the values of BF, Taq, Tth, Tfl, and Bsu (Table 1).

**Table 1:** The steady-state *Michaelis* constant (*K*_m_) of 3’-Aep-dCTP for commercially available A-family DNA polymerases.

DNA Polymerase	*K*_m_ (μM)
BF	1.50 ± 0.02
KF	0.28 ± 0.01
Taq	1.04 ± 0.19
Tth	1.45 ± 0.07
Tfl	1.63 ± 0.09
Bsu	2.09 ± 0.25

The NT-incorporation activity of all four 3’-Aep-dNTPs by BF and KF were further examined in more details. As shown in the Table 2, KF shows more robust NT-incorporation activities with each of the 3’-Aep-dNTPs than BF under the identical experimental conditions. The *K*_m_ values of KF for all four 3’-Aep-dNTPs are 0.24 ± 0.01 (dA), 0.08 ± 0.01 (dT), 0.28 ± 0.01 (dC), and 0.20 ± 0.01 μM (dG), respectively, which are all lower than the values of BF [0.95 ± 0.07 (dA), 0.20 ± 0.01 (dT), 1.50 ± 0.02 (dC), and 0.54 ± 0.01 μM (dG), respectively]. Among all four 3’-Aep-dNTPs, the BF shows a strong preference for the 3’-Aep-dTTP and efficiently incorporates this nucleotide. On the contrary, the 3’-Aep-dCTP is the least efficient nucleotide used for DNA replication by BF. Although KF also shows a higher incorporation activity with the 3’-Aep-dTTP, it has less bias for the other three 3’Aep-dNTPs (Table 2).

**Table 2:** The steady-state *Michaelis* constant (*K*_m_) of 3’-Aep-dNTP for BF and KF.

3’-Aep-dNTP	*K*_m_ (μM)	
	BF	KF
3’-Aep-dATP	0.95 ± 0.07	0.24 ± 0.01
3’-Aep-dTTP	0.20 ± 0.01	0.08 ± 0.01
3’-Aep-dCTP	1.50 ± 0.02	0.28 ± 0.01
3’-Aep-dGTP	0.54 ± 0.01	0.20 ± 0.01

### Faithful incorporation of 3’-Aep-dNMPs by BF and KF

**Figure 4:**
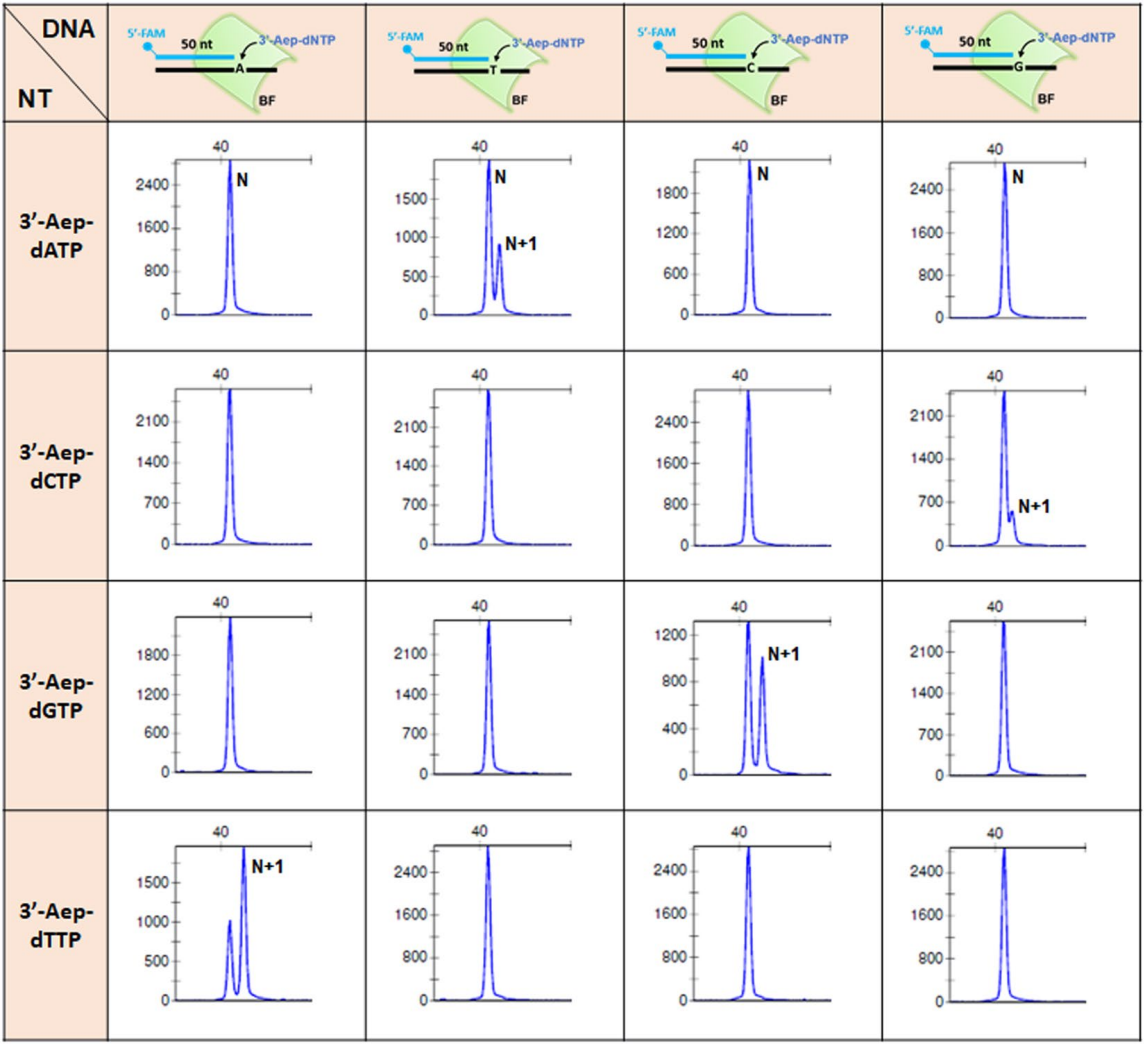
Selectivity of correct verse incorrect 3’-Aep-dNTP (Oligo_T#1~4) by BF. Each reaction contains 3 nM of DNA primer (Oligo_P#1), 1 μM of 3’-Aep-dNTP and 0.5 U of BF. The assay was performed at 60 °C for 5 minutes as described in the Methods. The primer (N) and the primer plus an incorporated 3’-Aep-dNMP by BF (N + 1) are indicated.

To accommodate the 3’-Aep-dCTP, our modeling suggests that BF has to adapt an alternative conformation in the nucleotide-binding pocket (NBP), mainly at the E658 sugar-steric gate and the O-helix of finger subdomain (Fig. 2C). The subtle structure shifts may alter the active-site geometry in the closed conformation of BF ternary complex and affect its nucleotide (NT) selectivity^33^. To evaluate whether the 3’-Aep-dNTP-induced structural changes may alter the selection of correct and incorrect NT by BF during DNA replication, the primer-extension assays were performed followed by DNA fragment analysis. As shown in the Fig. 4, the BF selectively incorporated the corresponding complementary nucleotide (primer + dNMP) opposite the templating base. Similar results were obtained when a 1 mM concentration of each 3’-Aep-dNTP (approximately 1,000-fold higher than the *K*_*m*_ values of each nucleotide) were used in the same reaction conditions (data not shown). Likewise, when the KF was tested under the same experimental conditions, the correct, complementary 3’-Aep-dNMP was selectively incorporated into the DNA (Figure S3). Hence, both BF and KF maintain a good NT selectivity for 3’-Aep-dNTPs during DNA replication.

### The 3’-esterified group of 3’-Aep-dNMP is edited during the nucleotide incorporation by BF

**Figure 5:**
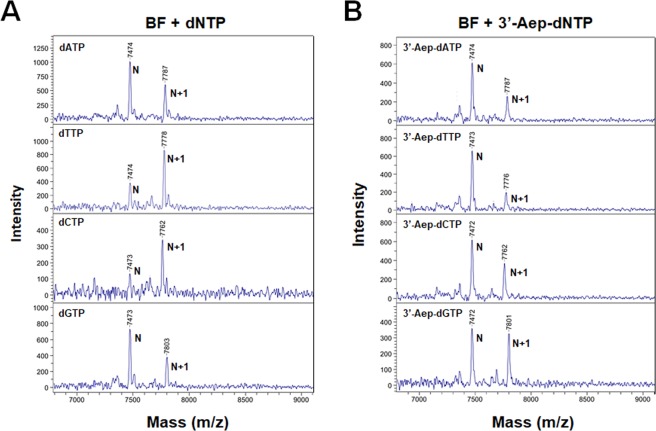
Analysis of incorporated 3’-Aep-dNMP by BF using the MALDI-TOF mass spectrometry. Each reaction mixture contains 0.2 μM of DNA primer (Oligo_P#2), 10 μM of dNTP or 20 μM of 3’-Aep-dNTP, and 1 U of BF. Each reaction was performed at 60 °C for 15 minutes as described in the Methods. The mass of primer (N) and the primer plus an incorporated dNMP or 3’-Aep-dNMP by BF (N + 1) were labeled next to the corresponding peaks, respectively.

Two A-family DNA polymerases (AF-DNAPs), a 3’→5’ exonuclease-deficient T7 (Sequenase) and Taq were previously shown to productively use various 3’-esterified dNTPs for DNA replication^25,26,29^. However, not every modification of 3’-esterified nucleotides can prevent the growing DNA-chain from further elongation by AF-DNAPs. The bulky 2’-deoxyl-3’-anthranyloyl-dNTP acts as a DNA chain-terminator once it is incorporated into the DNA by Taq^25,29^. Conversely, the incorporated 3’-O-acylated dNMP cannot completely block the DNA extension by the same enzyme (Taq), because the 3’-O-acylated group of NT is removed during the DNA replication^26^. Therefore, we would like to examine whether the (2-aminoethoxy)-3-propionyl group of 3’-Aep-dNMP remains intact after it is incorporated into the nascent DNA chain by BF. The single-nucleotide incorporation reactions by BF were performed in the primer-extension assays using an either natural dNTP or 3’-Aep-dNTP. The reaction products were then applied for the Matrix-Assisted Laser Desorption/Ionization-Time of Flight Mass Spectrometry (MALDI-TOF MS) analysis as described in the Methods. The molecular mass and identity of primer (N) or primer plus a nucleotide addition (N + 1) were determined. As shown in the Fig. 5, the primer-extension reaction products with an either natural dNTP (**Left panel**) or 3’-Aep-dNTP (**Right panel**) shows identical, corresponding mass for both the primer (m/z 7,474) and primer plus an addition of dAMP (m/z 7,787), dTMP (m/z 7,778), dCMP (m/z 7,762), or dGMP (m/z 7,803), respectively. These results indicate that the (2-aminoethoxy)-3-propionyl group of 3’-Aep-dNMP has been hydrolyzed back to a normal 3’-OH after it is incorporated into the growing DNA chain by BF.

### The enzyme-mediated deprotection of 3’-Aep-dNMP permits multiple nucleotide incorporations by BF on homopolymeric DNA templates

**Figure 6:**
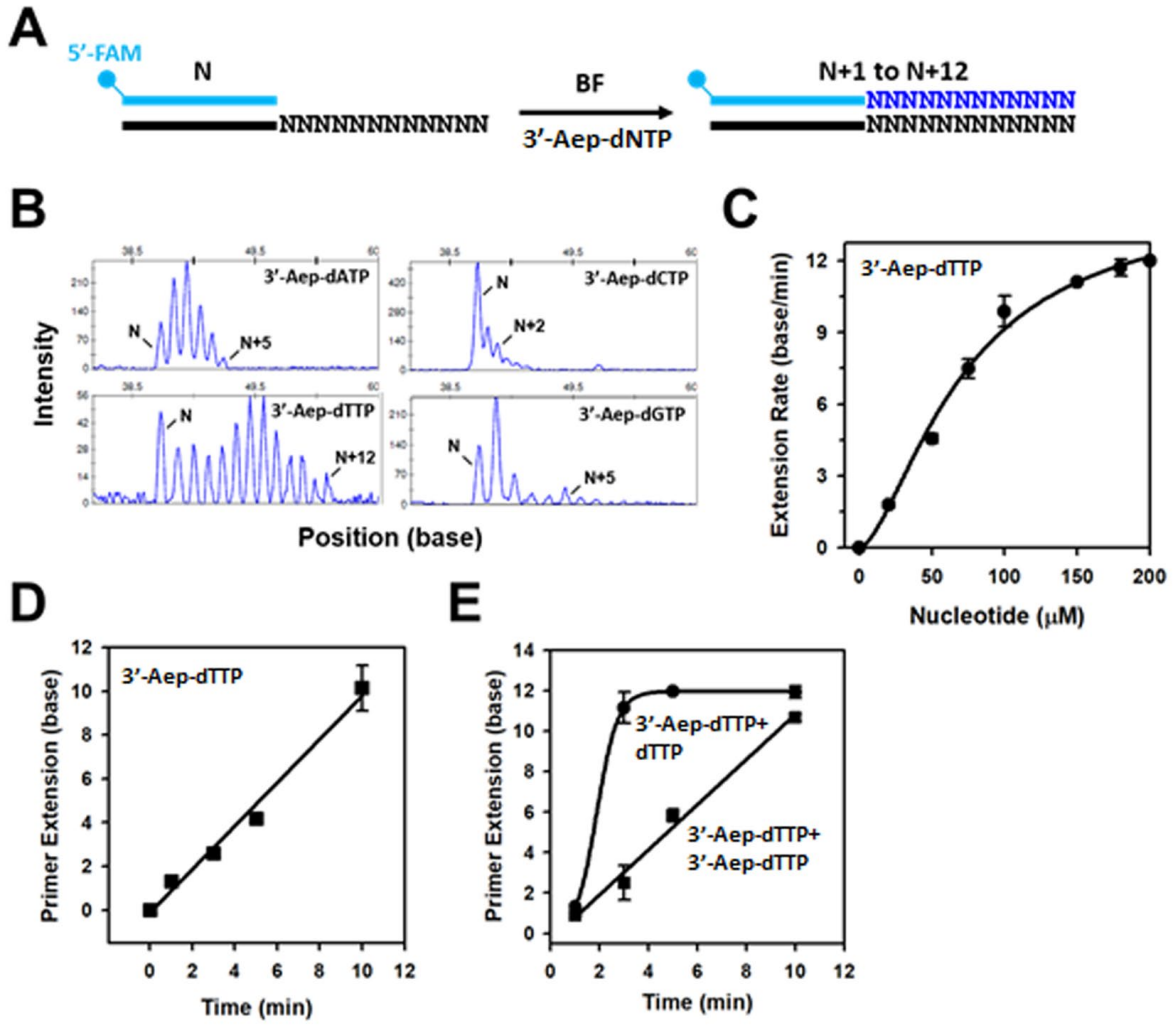
Multiple incorporations of 3’-Aep-dNTP by BF on a homopolymeric DNA template. (**A**) Schematic representation of primer-extension reaction across a homopolymeric DNA template by BF in the presence of matched 3’-Aep-dNTP (10 μM). (**B**) Continuous incorporations of 3’-Aep-dNMP by BF on the corresponding homopolymeric DNA template (Oligo_T#9~12). (**C**) The primer-extension rate of BF on the dA_12_ homopolymeric template (Oligo_T#9) in the presence of 25, 50, 75, 100, 150, 175, and 200 μM of 3’-Aep-dTTP. The primer-extension rate at each nucleotide concentration was calculated as described in the Methods. (**D**) Time course of multiple 3’-Aep-dTMP incorporations by BF across a dA_12_ homopolymeric template in the presence of 10 μM 3’-Aep-dTTP. (**E**) Results of 3’-Aep-dTMP incorporations followed with 10 μM of dTTP or 3’-Aep-dTTP chasing for 1 min.

The 3’-substituted group of 3’-Aep-dNTP is stable in the presence or absence of BF under the assay conditions (Figure S4). Additionally, the heat-inactivated BF loses both the DNA polymerization and 3’-esterase activities (Figure S5). Hence, it is likely that the (2-aminoethoxy)-3-propionyl group of 3’-Aep-dNTP is modified and restored back to a regular 3’-OH by BF during DNA replication. In any case, after each round of 3’-Aep-dNMP incorporation by BF, the 3’-terminus of DNA primer is readily converted back to a normal 3’-OH. Hence, multiple 3’-Aep-dNMP incorporations by BF should happen in the primer-extension reaction. To test whether BF would consecutively incorporate multiple 3’-Aep-dNMPs during DNA synthesis, the primer-extension assays were performed using either a homopolymeric dA-, dT-, dC-, or dG-DNA template in the presence of a corresponding, matched 3’-Aep-dNTP, respectively. The DNA template contains a twelve dA, dT, dC, or dG-sequence repeat (dN_12_) as depicted in the Fig. 6A. The reaction products after each 3’-Aep-dNTP addition show a stretch of continuous, elongated DNA peaks, which represent sequential 3’-Aep-dNMP incorporations by BF ranging from 1 to 12-NT insertions on the corresponding homopolymeric DNA template (Fig. 6B). Under the steady-state reaction conditions, the distance of extended DNA fragments or peaks from the original primer position is time-dependent. The longer the reaction time corresponds to the farther migration of extended DNA peaks (Fig. 6D). Moreover, the overall time required for BF to replicate across the twelve NT-repeat (dN_12_) region relies on the concentrations of 3’-Aep-dNTP in the reaction. A higher concentration of 3’-Aep-dNTP in the reaction drives a faster DNA elongation by BF across the homopolymeric DNA template (Fig. 6C).

Next, we examined whether the DNA primer with an incorporated 3’-Aep-dNMP can be readily extended by a normal dNTP on the homopolymeric dN_12_-DNA template. The primer-extension assays were performed in a stepwise manner by initially adding a 3’-Aep-dNTP in the reaction followed with the supplementation with a cognate, natural dNTP or the same 3’-Aep-dNTP. As shown in the Fig. 6E, the DNA primer was rapidly extended through the homopolymeric dA_12_-template by BF right after chasing with the normal dTTP. In contrast, an extra 3’-Aep-dTTP addition in the reaction didn’t influence the speed of primer extension by BF. These results confirm that the 3’-substituted group of 3’-Aep-dNMP is changed back to a *bona fide* 3’-OH after it is added into a growing DNA chain.

### The cleavage of the 3’-protective group of incorporated 3’-Aep-dNMP by BF from the 3’-primer terminus enables a long, continuous DNA synthesis

**Figure 7:**
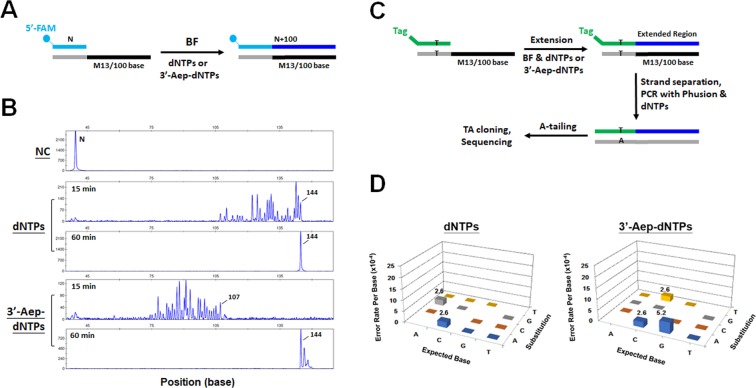
Continuous DNA synthesis of BF using all four 3’-Aep-dNTPs. (**A**) Schematic representation of DNA elongation assay using the bacteriophage M13-DNA as a template (Oligo_T#13) together with all four dNTPs or 3’-Aep-dNTPs. (**B**) Time course of DNA elongation by BF with 20 μM of dNTPs or 3’-Aep-dNTPs, respectively, in the reaction. (**C**) Method to determine the base-substitution errors of BF in the presence of normal dNTPs or 3’-Aep-dNTPs. (**D**) The replicative error profile of BF in the presence of normal dNTPs (**Left**) or 3’-Aep-dNTPs (**Right**). The expected, or correct, A, T, C or G base, respectively, are listed on the x-axis. The actual base (A, T, C, or G) change identified are represented in the y-axis. The z-axis shows the frequency of correct base gets substituted.

Because the 3’-ester modification of 3’-Aep-dNMP is concurrently removed from the 3’-end of the growing DNA chain after each cycle of NT addition by BF, it should permit an uninterrupted DNA replication. To further test whether BF would use all four 3’-Aep-dNTPs for a long, continuous DNA synthesis, the primer-extension assays were carried out using a fluorescein (FAM)-labeled DNA primer (P) pre-annealed with a 100 nucleotide-long-DNA template in the presence of all four dNTPs or 3’-Aep-dNTPs (Fig. 7A). As shown in the Fig. 7B, a time-dependent DNA elongation by BF was observed after the addition of either dNTPs or 3’-Aep-dNTPs, while a single DNA-primer peak (P) was seen in the negative control (NC) reaction in the absence of nucleotides (**Top panel**). The elongated DNA products generated by BF with 3’-Aep-dNTPs showed a distribution of various DNA fragment sizes between ~75 and 110 nucleotides (NTs) after 15 minutes of reaction. On the other hand, longer lengths of DNA fragments, ranging from ~106 to 144 NTs, were observed when the normal dNTPs were present in the same experimental conditions. Nonetheless, the primer-extension reactions with either 3’-Aep-dNTPs or natural dNTPs were both completed within an hour of reaction. Evidently, the inserted 3’-Aep-dNMPs do not prevent the DNA extension by BF and permit a long, continuous DNA synthesis.

Finally, we would like to learn whether the utilization of all four 3’-Aep-dNTPs by BF maintains a good accuracy of DNA replication. To determine the error profiles of DNA synthesis by BF, DNA primers containing unique DNA sequence tags at the 5’-end were used in the primer-extension reactions as illustrated in the Fig. 7C. The extended DNA products were further amplified by PCR, cloned into a TA-cloning vector, and transformed into the *E. coli* cells. The recombinant vectors harboring the BF-replication DNA fragments were recovered from individual bacterial colonies and then sequenced. The base-substitution errors of DNA replication by BF were analyzed. As shown in the Fig. 7D, the overall replicative fidelity of BF in the presence of 3’-Aep-dNTPs are equivalent to the values of natural dNTPs. However, the DNA synthesis reaction with the 3’-Aep-dNTPs show slight elevations of C → T and G → A transition mutations to 2.6 ×10^-4^ and 2.6 ×10^-4^ errors/base, respectively, above the background value. Interestingly, the frequency of A → G transition mutations in the 3’-Aep-dNTPs reaction was lower than that in the natural dNTP reaction. Overall, the BF can utilize the 3’-Aep-dNTPs for a faithful and unceasing DNA replication.

## Discussion

In this study, we synthesized all four (2-aminoethoxy)-3-propionyl (Aep)-dNTPs and examined the nucleotide-incorporation activities by six AF-DNAPs (BF, KF, Taq, Tth, Tfl, and Bsu), respectively. We also showed that the KF and BF efficiently and faithfully incorporated 3’-Aep-dNMPs. Additionally, we analyzed the post-incorporation products of N + 1 primer by BF and confirmed the (2-aminoethoxy)-3-propionyl group of 3’-Aep-dNMP was modified back to a regular 3’-OH after it was inserted into the growing DNA chain. By applying all four 3’-Aep-dNTPs for an *in vitro* DNA synthesis reaction, we demonstrated that the A-family DNA polymerases can efficiently insert 3’-Aep-dNMPs and permit a long, continuous, and scar-free DNA synthesis.

### The highly conserved motif-A and -B residues of A-family DNA polymerase play a critical role in accommodating the 3’-esterified dNTP

High resolution crystal structures of KF^34^, Taq^35^, T7^36^, and BF^33^ DNA polymerases all show that these A-family DNA polymerases (AF-DNAPs) undergo a structural transition from an open to a closed state upon binding with an incoming nucleotide (NT). The NT binding coupled with the DNA polymerase conformational change establishes multiple pre-chemistry NT-selection checkpoints to ensure a correct NT binding inside the nucleotide-binding pocket (NBP)^37,38^. The closed conformation of DNA polymerase significantly confines the geometry of NBP and, thus, limit (or select for) the shapes and configurations of the incoming NT to form a proper, complementary Watson-Crick base pair with the N + 1 templating base. Recent single-molecule studies have demonstrated that a smooth transition of KF from an open to a “fully” closed conformational state assure a high fidelity of DNA synthesis by DNA polymerase^38^.

In the closed ternary complex of BF, the conserved palm and the O-helix of finger subdomains define the boundaries of nucleotide-binding pocket (NBP) for interrogating the incoming nucleotide^33^. At the bottom of NBP, the side-chain carboxylate of the highly conserved E658 on the motif A of the palm subdomain (Fig. 2B) serves as a “sugar-steric gate” to exclude the 2’-OH of ribose of the NT. Therefore, the elimination of this side-chain carboxylate of E658 by substituting the glutamate with alanine (E658A) greatly reduces the ribonucleotide (rNTP) discrimination during DNA replication. As a result, the mutant BF^E658A^ notably misincorporates the rNMP during DNA synthesis^33^. Besides the steric gate function, the side chain carboxylate of E658 also forms a direct hydrogen bond with the 3’-OH of nucleotide (NT) and coordinates the alignment of NT with the 3’-OH of primer terminus in the pre-chemistry step. A misalignment of active site residues with the NT and the primer terminus is observed in the ternary structure of the mutant BF^E658A^ in complex with the primer-template DNA and a nucleotide^33^. Furthermore, the mutant BF^E658A^ has a reduced nucleotide-binding affinity and overall incorporation activity^33^. In the current structural simulation of BF with the 3’-Aep-dNTP, the 3’-OH of normal dNTP is replaced by the 3’-ester carbonyl group of 3’-Aep-dNTP, which also forms a hydrogen bond with the side-chain carboxylate of E658 within the nucleotide-binding pocket (NBP) (Fig. 2D). The formation of this alternative hydrogen bond probably retains a proper arrangement of active-site residues in the NBP and the correct alignment of 3’-Aep-dNTP with the 3’-terminus of the primer. Hence, we hypothesize that the direct hydrogen bonding between the side-chain carboxylate of E658 and the 3’-OH of the incoming nucleotide (NT) might explain why the A-family DNA polymerases (AF-DNAPs) productively utilize the 3’-esterified NT, but not other types of 3’-modified NTs, for DNA replication^10,27^.

The upper boundary of NBP for restraining the incoming NT in the A-family DNA polymerase is delineated by the O-helix of finger subdomain, which consists of an evolutionally conserved motif B region. The O-helix of finger subdomain in KF has been shown to function as a fidelity checkpoint for scrutinize the shapes of nucleotide and the adequate geometry of nascent N + 1 base pair^34^. The protein sequence alignment among AF-DNAPs indicates that this motif B region contains a consensus sequence –RRxhKhhN**F**Ghh**Y**– **(**Fig. 2B**)**, in which the h and x represent a hydrophobic and any type of amino acid, respectively. Structurally, the phenylalanine (F710) and tyrosine (Y714) residues in the motif B region locate on the same side of the O-helix and face toward the nucleotide-binding pocket (NBP) of BF (Fig. 2C). In the presence of nucleotide (NT), the NT binding induces a ≥ 40° rotation (closing) of the O-helix toward the palm subdomain. The closing of the O-helix brings the planar aromatic side-chains of both F710 and Y714 to directly contact and check the configurations of the base and deoxyribose of nucleotide inside the NBP (Fig. 2C). In our computational model of BF with the 3’-Aep-dCTP, both F710 and Y714 slightly re-adjust their positions to accept the 3’-Aep-dCTP in the NBP. The repositions of F710 and Y714 may secure the NT binding inside the NBP and promote the subsequent NT-incorporation by BF. Furthermore, the biased 3’-Aep-dNMPincorporation by BF **(**Fig. 4**)** may stem from the altered base-pair geometries of the nucleotide-binding pocket, which may be caused by different conformational states of the finger subdomain as previously suggested^39–41^.

In summary, the alternative hydrogen bonding and stacking interactions between the conserved motif A and B residues of A-family DNA polymerase (AF-DNAP) and the 3’-Aep-dNTP may greatly stabilize the nucleotide binding and nascent N + 1 base-pairing geometry inside the nucleotide-binding pocket. Consequently, the 3’-Aep-dNTP is permitted to pass through multiple fidelity checkpoints in the closed conformation of AF-DNAP. In return, the AF-DNAP can accurately and efficiently incorporate the 3’-Aep-dNMP during DNA synthesis.

### The mechanism of DNA polymerase-mediated cleavage of the inserted 3’-esterified nucleotide remains unknown

The 3’-esterase activity of A-family DNA polymerase (AF-DNAP) was originally reported by Sarfati and coworkers in 1995^29^. However, it remains unclear how the AF-DNAP hydrolyzes the 3’-ester substitution from the incorporated 3’-esterified nucleotide. In Sarfati’s earlier report, the 3’-catalytic editing function of Taq DNA polymerase on the 3’-esterified nucleotide (NT) or primer depends on the presence of the next, correct incoming NT relative to the templating base. Using a 3’-anthranyloyl (3’-ant) protecting NT or primer-template DNA in the primer-extension assay, the removal of 3’-blockage group from the NT or 3’-primer terminus by T7 DNA polymerase only occurs when the incoming NT correctly base-paired with the templating base^29^. Our current results are in a good agreement with Sarfati’s study. Additionally, our data indicate that the removal of 3’-esterified group from the 3’-primer terminus limits the rate of next correct nucleotide addition. In the nucleotide-chasing experiments, the primer extension activity of BF across the homopolymeric DNA template is much more robust right after chasing with the normal dNTP, but not the 3’-Aep-dNTP (Fig. 6E). To put things into perspective, we further hypothesize that the DNA polymerization reaction should proceed before the hydrolysis of the 3’-esterified substitution of the inserted NT. Moreover, our study also strongly supports the Sarfati’s view that the intrinsic 3’-esterase and DNA polymerase activity of AF-DNAP may share the same catalytic center^29^.

### Potential application of 3’-esterified nucleotides for a scar-free, continuous DNA sequencing-by-synthesis reaction

In the conventional reversible dye-terminator (RDT)-based Sequencing-by-synthesis (SBS) chemistry, each reaction cycle generates unnatural, chemical residues on the newly synthesized DNA^10,20,21^. To avoid the chemical scar issue in the current RDT-based SBS chemistry, we propose to use the A-family DNA polymerase (AF-DNAP) and 3’-esterified nucleotide (NT) directly attached with a fluorescent dye via the 3’-ester modification of the NT (designated as a 3’-FT-NT) for SBS reaction as illustrated in the Fig. 1 **(Bottom reaction pathway)**. In the preliminary study, we observed that BF can efficiently incorporate the 3’-Aep-dNMP attached with an ATTO532 fluorescent dye (3’-Aep-ATTO532-dNTP, Figure S6). Our novel chemistry can potentially be further tested for a scar-free DNA sequencing reaction.

## Methods

### Chemicals and enzymes

All chemicals were purchased from Sigma-Aldrich unless otherwise indicated. Oligonucleotides, used as primers or templates, were commercially synthesized (Mission Biotech, Taipei, Taiwan). The sequences of oligonucleotides were listed in the Table S1. The 3’-Aep-dNTPs were designed and synthesized in-house (Personal Genomics, Hsinchu, Taiwan). The fluorescent dye-labeled 3’-Aep-ATTO532-dATP was produced via the direct conjugation of the ATTO532 fluorophore to the Aep-linker at the 3’-O position of deoxyribose. The molecular mass, identity, purity, and stability of each modified nucleotides were evaluated by the ESI-MS (Figure S1) and HPLC (Figure S2), respectively. BF, Bsu, Taq, and KF DNA polymerases were obtained from New England Biolabs (Ipswich, MA). Tfl and Tth DNA polymerases were purchased from Promega (Madison, WI). DNA polymerases were initially titrated and optimized in the reaction, which included 0.5U of BF, 0.1U of Taq, 0.1U of Tfl, 0.1U of Tth, 0.1U of Bsu, or 0.1U of KF, respectively.

### Structural modeling

The ternary structural model of BF with a normal dCTP or a modified 3’-Aep-dCTP were simulated according to the previously published method^42^. The BF ternary structure [PDB ID: **1LV5**]^31^ is used as a structural template and its active site was aligned with a bound nucleotide (NT) using the pair-fit function in PyMol (Schrödinger, LLC)^43^. The modelled structures were then energy minimized using BIOVIA Discovery Studio 4.5 (BIOVIA, San Diego, CA). The maximum of 1,000 steps were set with minimization RMS Gradient tolerance of 0.1 kcal/(mol × Å) to terminate the minimization routine in case the average gradient is less than (or equal to) the set cut-off, thus providing the simulated model.

### Primer-extension assay

DNA oligonucleotides used in the primer-extension (PE) assays are listed in the Table S1. The primers used for the nucleotide incorporation and DNA elongation assays were all 5’-labeled with a 5’-6-carboxyfluorescein (FAM) for signal detection. The 5’-FAM-labeled primers were individually annealed with a complementary DNA template in 1X ThermoPol buffer [20 mM Tris-HCl, pH 7.5, 10 mM (NH_4_)_2_SO_4_, 10 mM KCl, 2 mM MgSO_4_, and 0.1% Triton X-100] before mixing with nucleotides and DNA polymerase. In the PE reaction, the indicated amount of DNA polymerase was pre-incubated with the primer/template DNA on ice for 3 minutes before the addition of nucleotide(s). The reactions were performed at 60 °C for 10 minutes, and then quenched with 1.25 μl of stop solution (0.5 mM EDTA)^30^. The PE products were separated by capillary electrophoresis (CE) on an ABI 3500 genetic analyzer (Applied Biosystems, Foster City, CA) using the POP-7 polymer and 36-cm length of capillary. The capillary gel electrophoresis and sample were prepared as previously described^30,44^. The CE data were analyzed by GeneMapper v4.0 and displayed as a linear-log plot of product formation in response to different nucleotide concentrations. The average primer-extension rate (*r*) of BF on the homopolymeric DNA template at each nucleotide concentration (Fig. 6C) was estimated by the following procedure: 1) the intensity of each peak (P_N_) from the electropherogram was first multiplied by the number of bases (N) got extended from the primer position. The sum of all P_N_ × N was then divided by the sum of total (P_N_ + P_0_). P_0_ represents the peak intensity of original primer. Since the reaction time was fixed at one minute, the average primer-extension rate (bases/minute) of BF on the homopolymeric DNA template at each nucleotide concentration was then determined.

### MALDI-TOF/TOF MS analysis

The analysis of post-insertion DNA products were performed as described previously with the Bruker AutoFlex III smartbeam TOF/TOF 200 system (Bruker Daltonics, MA)^30,45^. The primer and template DNA sequences used in the assays are listed in Table S1. The primer was pre-annealed with the template DNA at a 1:1.2 ratio in 10× reaction buffer containing 100 mM KCl, 20 mM MnCl_2_, and 100 mM Tris-HCl, pH 7.5. In the PE assay, the DNA polymerase was pre-incubated with the duplex P/T-DNA on ice for 3 minutes. Then, the 3’-Aep-dNTP was added to initiate reaction, and the reaction mixture was incubated at 60 °C for one hour. The reaction was quenched with 2 μl of acetonitrile. Before the MS analysis, the reaction mixture was first cleaned up with Micro Bio-Spin 30 column (Bio-Rad, CA). The column-eluted sample (0.5 μl) was mixed with a 0.5 μl of 3-HPA (3-hydropicolinic acid) matrix in acetonitrile. The sample was then dried and analyzed. The positive ion mode was applied to collect all spectra.

### DNA elongation assay

The sequences of duplex primer-template (P/T)-DNA substrates used in the assay are listed in Table S1. The reactions were performed at 60 °C for one hour by mixing the DNA polymerase with the P/T-DNA substrate and nucleotides in 1x reaction buffer containing 10 mM (NH_4_)_2_SO_4_, 10 mM KCl, 5 mM MgSO_4_, 20 mM Tris-HCl (pH 7.5), and 0.1% Triton X-100. The analysis of elongated DNA products was performed as described in the PE assay^30,44^.

### Error profiling of BF DNA Replication

The fidelity of BF DNA replication in the presence of normal dNTPs or 3’-Aep-dNTPs were evaluated as described previously^30,46^. Briefly, the primer-extension (PE) assays were performed using a DNA primer pre-annealed with a M13 phage DNA template (Table S1). The reaction was performed at 60 °C for one hour and the resulting products were purified by Qiagen PCR clean-up kit. The purified PE products were further amplified by a High-Fidelity Phusion DNA Polymerase (ThermoFisher Scientific, Waltham, MA) according to the manufacturer’s instructions. The amplified PCR products were A-tailed by Taq DNA polymerase (NEB). The A-tailing products were purified by Qiagen PCR clean-up kit, and then ligated with the TA cloning vector (Yeastern Biotech, Taipei, Taiwan) following the manufacturer’s protocol. The ligation mixture was transformed into *Escherichia coli* DH5α cells. Individual colonies were grown in the LB broth and directly submitted for DNA sequencing service (MB Biotech, Taipei, Taiwan). DNA sequences were aligned with the M13 phage DNA template and analyzed by BioEdit 7.0 (IIbis Therapeutics, Carlsbad, CA). DNA sequences lacking a T to A watermark were discarded as they were generated from the starting DNA template rather than replicated materials.

## Supplementary information

Supplementary Information.
