# Supplementary material for: Enzymatic Cleavage of 3’-Esterified Nucleotides Enables a Long, Continuous DNA Synthesis
Source: Sci Rep. 2020 May 5;10:7515. doi: 10.1038/s41598-020-64541-z (PMC7200780; doi:10.1038/s41598-020-64541-z)
Supplement: Supplementary file 1 — Supplementary Information. [file 41598_2020_64541_MOESM1_ESM.pdf]

Enzymatic Cleavage of 3'-Esterified Nucleotides Enables a Long, Continuous DNA Synthesis

Shiuan-Woei LinWu[1], Ting-Yueh Tsai[1], Yu-Hsuan Tu[1], Hung-Wen Chi[1], Yu-Ping Tsao[1], Ya-Chen Chen[1], Hsiang-Ming Wang[1], Wei-Hsin Chang[1], Chung-Fan Chiou[1], Johnsee Lee[1], and Cheng-Yao Chen[2,3*]

[1]Personal Genomics, Inc., Zhubei, Hsinchu 30261, Taiwan

[2]Department of Medical Laboratory Science and Biotechnology, College of Medicine, National Cheng Kung University, Tainan City 70101, Taiwan

[3]Institute of Biological Chemistry, Academia Sinica, Nankang, Taipei 115, Taiwan

*Correspondence should be addressed to Dr. Cheng-Yao Chen (chengyao@mail.ncku.edu.tw).

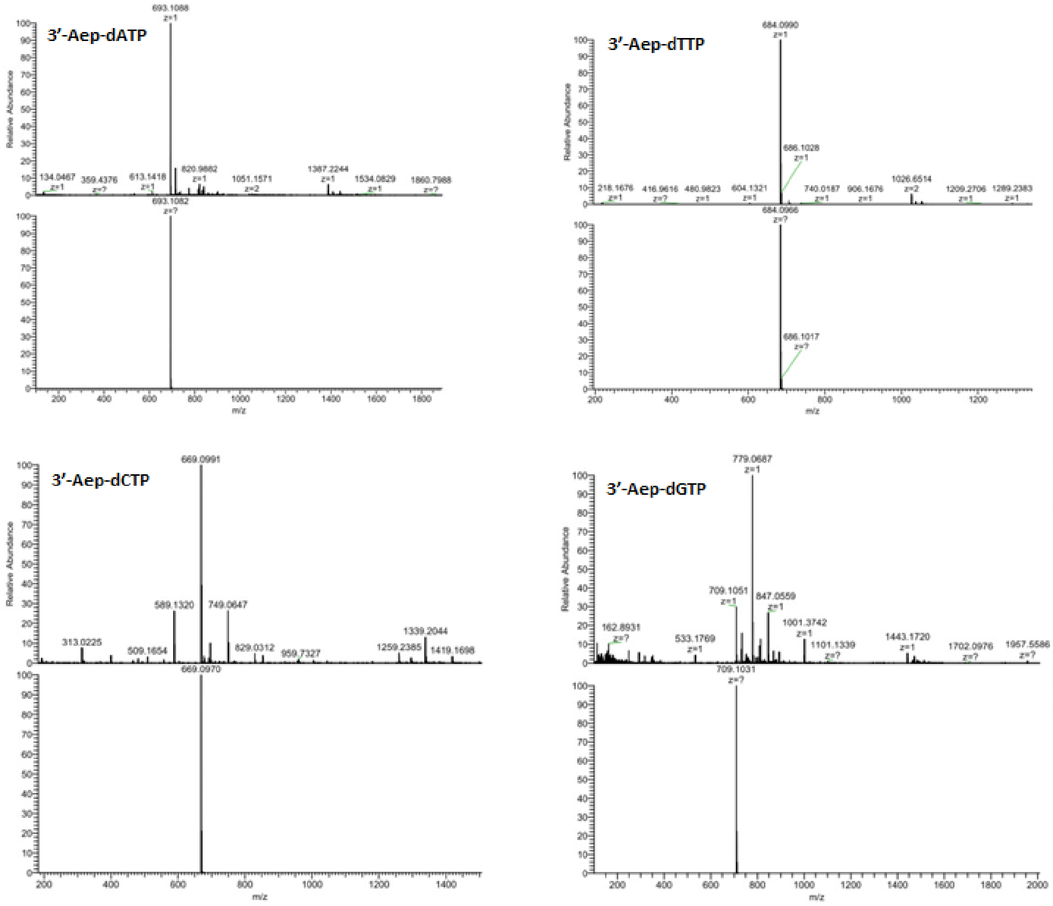

Figure S1. Analysis of 3'-Aep-dNTP using ESI-MS analysis.

The nucleotide analysis was performed as described previously with the Bruker AutoFlex III smartbeam TOF/TOF 200 system (Bruker Daltonics, MA)[30,45].

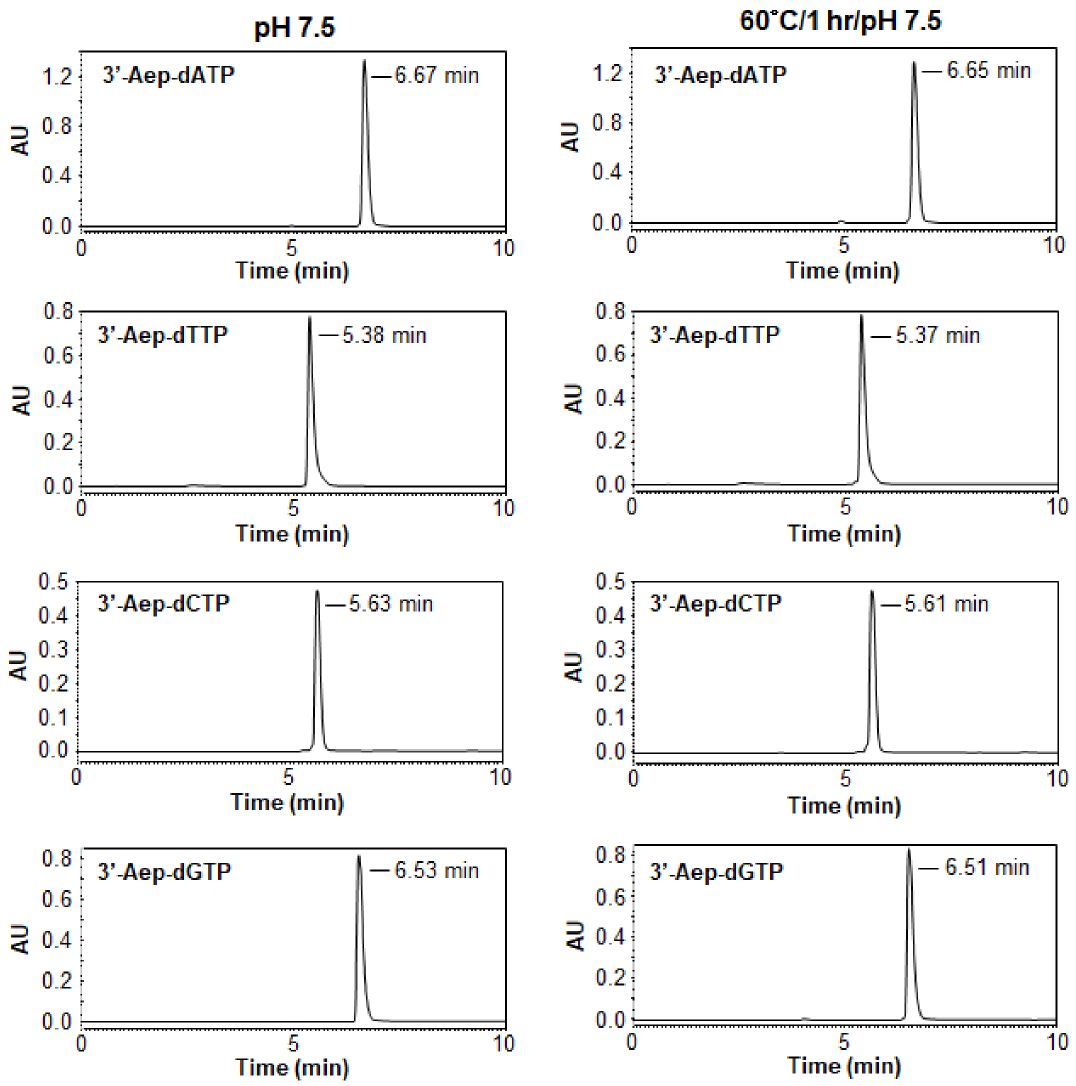

Conditions	Purity			
	3’-Aep-dATP	3’-Aep-dTTP	3’-Aep-dCTP	3’-Aep-dGTP
pH 7.5	99.32%	99.92%	99.50%	99.75%
pH 7.5/1 hr/60˚C	98.75%	99.57%	99.50%	99.33%

Figure S2. Purity and stability of 3’-Aep-dNTP verified by HPLC analysis.

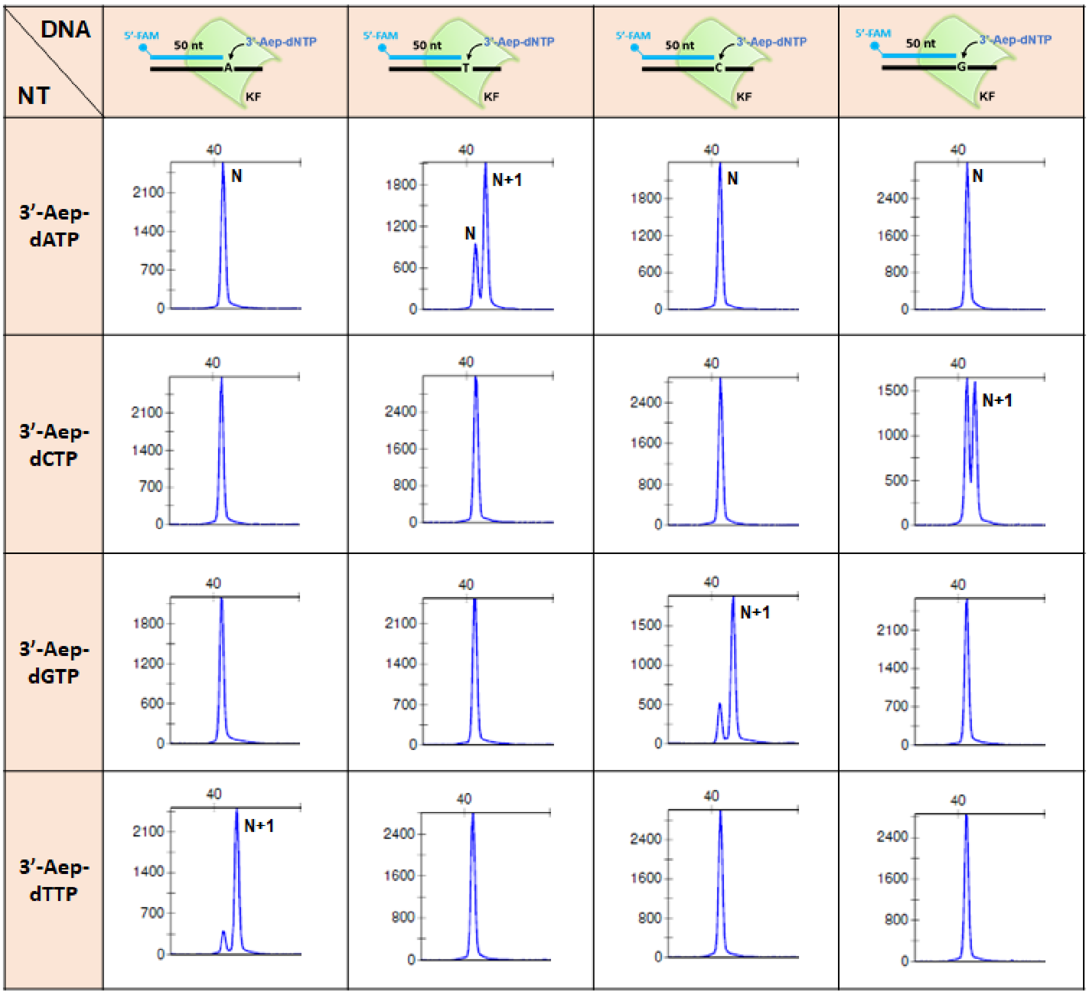

Figure S3. Selectivity of matched and mismatched 3'-Aep-dNTP by KF. Each reaction contains 3 nM of DNA primer, 1 μM of 3'-Aep-dNTP and 0.1U of KF as described in the Methods. Reactions were performed at 60°C for 5 minutes.

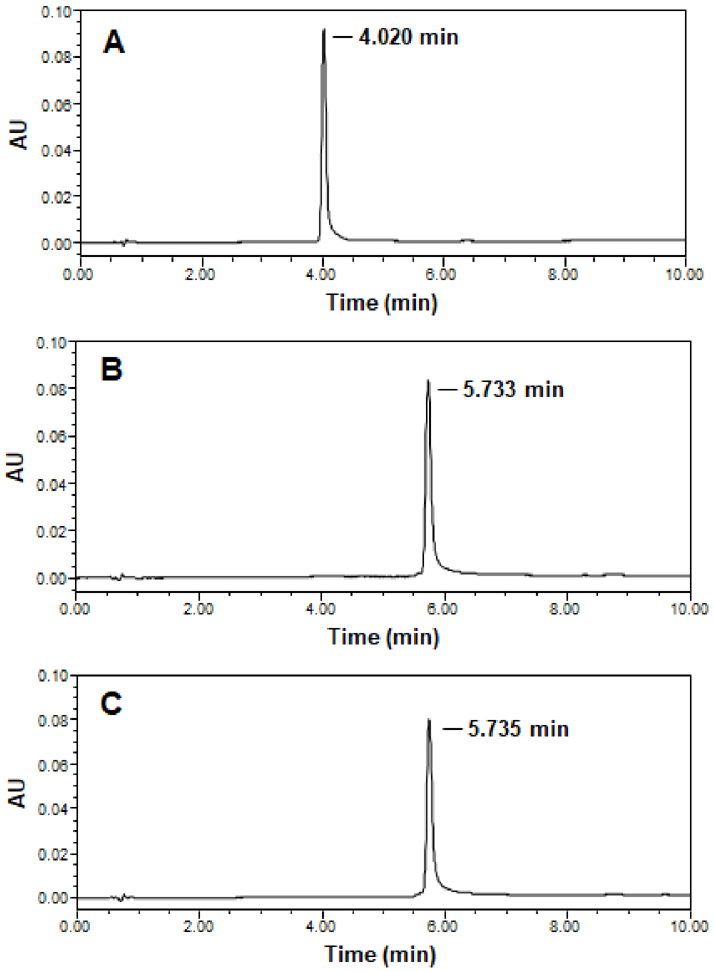

Figure S4. Analysis of 3’-Aep-dATP stability in the presence of BF in the DNA polymerase reaction condition by HPLC. (A) 80 μM of dATP only, (B) 80 μM 3’-Aep-dATP only, and (C) 80 μM of 3’-Aep-dATP and BF. The reactions were performed at 45°C for 10 minutes.

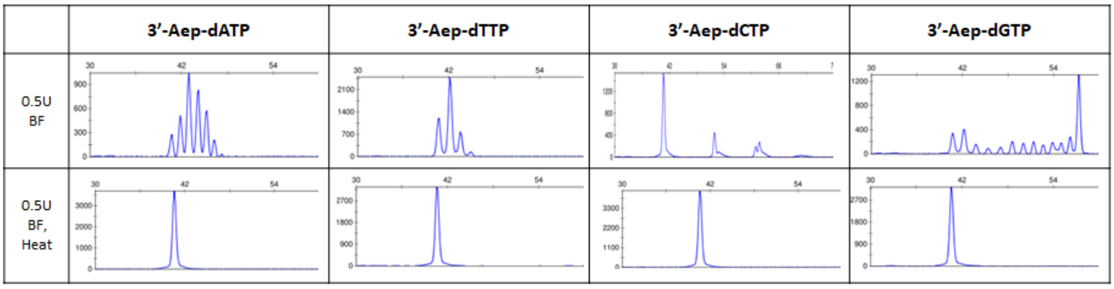

Figure S5. Multiple incorporations of 3'-Aep-dNMP by BF (Top) or heat-inactivated BF (Bottom) on the homopolymeric DNA template. The heat-inactivated BF was pre-treated at 90˚C for 15 minutes before added into the reaction mixture.

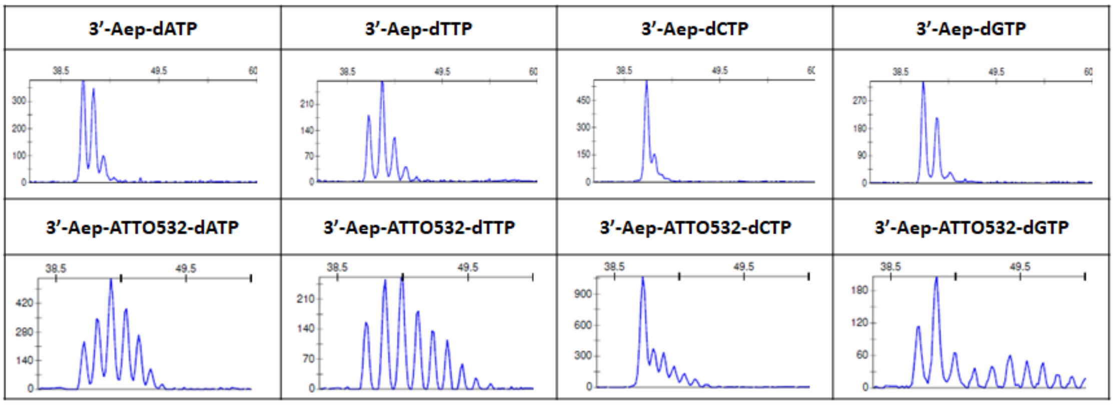

Figure S6. Multiple incorporations of 3’-Aep-dNMP and 3’-Aep-ATTO532-dNMP by BF on the homopolymeric DNA templates. All reactions were performed at 45°C for 30 seconds in the presence of 10 μM nucleotide.

Table S1. List of oligonucleotides used in this study[a].

No.	Sequence (5' to 3')	Experiment
Oligo_P#1	FAM-AGTGAATTCGAGCTCGGTACCCGGGGATCCTCTAGAGTCGACCTGCAGGC	PE & Kinetics
Oligo_T#1	TTGCTCGTTTGCTGGG**A**GCCTGCAGGTCGACTCTAGAGGATCCCCGGGTACCGAGCTCGAATTCACT	PE & Kinetics
Oligo_T#2	TTGCTCGTTTGCTAAA**G**GCCTGCAGGTCGACTCTAGAGGATCCCCGGGTACCGAGCTCGAATTCACT	PE & Kinetics
Oligo_T#3	TTGCTCGTTTGCTGGG**T**GCCTGCAGGTCGACTCTAGAGGATCCCCGGGTACCGAGCTCGAATTCACT	PE & Kinetics
Oligo_T#4	TTGCTCGTTTGCTGGG**C**GCCTGCAGGTCGACTCTAGAGGATCCCCGGGTACCGAGCTCGAATTCACT	PE & Kinetics
Oligo_P#2	CCCTCGCAGCCGTCCAACCAACTCA	MS
Oligo_T#5	TTTGTTCTCC**A**TGAGTTGGTTGGACGGCTGCGAGGG	MS
Oligo_T#6	TTTGTTCTCC**T**TGAGTTGGTTGGACGGCTGCGAGGG	MS
Oligo_T#7	TTTGTTCTGG**C**TGAGTTGGTTGGACGGCTGCGAGGG	MS
Oligo_T#8	TTTGTTCTCC**G**TGAGTTGGTTGGACGGCTGCGAGGG	MS
Oligo_T#9	TTACTCG**AAAAAAAAAAAA**GCCTGCAGGTCGACTCTAGAGGATCCCCGGGTACCGAGCTCGAATTCACT	PE of homopolymer
Oligo_T#10	TTACTCG**TTTTTTTTTTTT**GCCTGCAGGTCGACTCTAGAGGATCCCCGGGTACCGAGCTCGAATTCACT	PE of homopolymer
Oligo_T#11	TTACTTT**CCCCCCCCCCCC**GCCTGCAGGTCGACTCTAGAGGATCCCCGGGTACCGAGCTCGAATTCACT	PE of homopolymer
Oligo_T#12	TTACTTT**GGGGGGGGGGGG**GCCTGCAGGTCGACTCTAGAGGATCCCCGGGTACCGAGCTCGAATTCACT	PE of homopolymer
Oligo_P#3	FAM-CGAGCACGTATAACGTGCTTTCCTCGTTGGAATCAGAGCGGGAGCTAAAC	DNA elongation
Oligo_T#13	ATGGACAGACTCTTTTACTCGGTGGCCTCACTGATTATAAAAACACTTCTCAAGATTCTGGCGTACCGTTCCTGTCTAAAATCCCTTTAATCGGCCTCCTGTTTAGTCCCGCTCTGATTCCAACGAGGAAAGCACGTTATACGTGCTCG	DNA elongation
Oligo_P#4	CTTTTAAGAACCGGACGAACCGAGCACGT**T**TAACGTGCTTTCCTCG	Amplification for sequencing "**T**": watermark
Oligo_T#14	ATGGACAGACTCTTTTACTCGGTGGCCTCACTGATTATAAAAACACTTCTCAAGATTCTGGCGTACCGTTCCTGTCTAAAATCCCTTTAATCGGCCTCCTCGAGGAAAGCACGTTA**T**ACGTGCTCG	Amplification for sequencing "**T**": watermark
Oligo_P#5	CTTTTAAGAACCGGACGAACCGAG	Amplification for sequencing
Oligo_P#6	CTGGATATTACCAGCAAGGCCGAT	Amplification for sequencing

[a]The underlined sequences are complimentary regions, and the bolded bases are the templating bases for specific nucleotide incorporation.
